# Pollen Source Affects Development and Behavioral Preferences in Honey Bees

**DOI:** 10.3390/insects12020130

**Published:** 2021-02-02

**Authors:** Jun Lan, Guiling Ding, Weihua Ma, Yusuo Jiang, Jiaxing Huang

**Affiliations:** 1 College of Animal Science, Shanxi Agricultural University, Taigu 030801, China; lanj18235463638@163.com (J.L.); mawh1997@163.com (W.M.); 2 Key Laboratory for Insect-Pollinator Biology of the Ministry of Agriculture and Rural Affairs, Institute of Apicultural Research, Chinese Academy of Agricultural Sciences, Beijing 100093, China; dingguiling@caas.cn

**Keywords:** *Apis mellifera*, pear pollen, apricot pollen, hypopharyngeal gland, ovarian development

## Abstract

**Simple Summary:**

Pollinators adjust their foraging preference based on the pollen cues of foraging plants. Honey bees, for example, prefer to collect one type of pollen from plants that bloom at the same time. In northern China, apricot and pear trees are the two main foraging plants in the early spring. However, honey bees tend to collect pollen from apricot trees. It is interesting to understand what affects the foraging decision of honey bees regarding these two pollen types. In this study, we observed the foraging preference of *Apis mellifera* workers with respect to apricot and pear pollen under laboratory conditions. The effect of pollen on the development of the hypopharyngeal gland (HG) and ovary was measured. The number of visits made to apricot pollen was significantly higher than that to pear pollen. Furthermore, the response of the HG and ovary to these two pollens was different. The development of the HG was significantly affected by pollen diet treatments. However, there was no significant difference in the ovarian development of caged workers supplied with the two different pollen diets. Overall, honey bees showed a significant preference for apricot pollen over pear pollen. Compared with the ovary, the HG of honey bee workers may be more sensitive to pollen nutrition.

**Abstract:**

With the availability of various plants in bloom simultaneously, honey bees prefer to collect some pollen types over others. To better understand pollen’s role as a reward for workers, we compared the digestibility and nutritional value of two pollen diets, namely, pear (*Pyrus bretschneideri* Rehd.) and apricot (*Armeniaca sibirica* L.). We investigated the visits, pollen consumption, and pollen extraction efficiency of caged *Apis mellifera* workers. Newly emerged workers were reared, and the effects of two pollen diets on their physiological status (the development of hypopharyngeal glands and ovaries) were compared. The choice-test experiments indicated a significant preference of *A. mellifera* workers for apricot pollen diets over pear pollen diets (number of bees landing, 29.5 ± 8.11 and 9.25 ± 5.10, *p* < 0.001 and pollen consumption, 0.052 ± 0.026 g/day and 0.033 ± 0.013 g/day, *p* < 0.05). Both pollen diets had comparable extraction efficiencies (67.63% for pear pollen and 67.73% for apricot pollen). Caged workers fed different pollen diets also exhibited similar ovarian development (*p* > 0.05). However, workers fed apricot pollen had significantly larger hypopharyngeal glands than those fed pear pollen (*p* < 0.001). Our results indicated that the benefits conferred to honey bees by different pollen diets may influence their foraging preference.

## 1. Introduction

Bees and angiosperms have shared a long intertwined evolutionary history, and their interactions are excellent examples of mutualistic associations [1]. Pollination by honey bees is based on their foraging behavior, while foraged nectar is the main source of energy for adult bees, and pollen provides adult bees and larvae with protein, lipids, vitamins, and minerals [2]. Pollen is essential for the glandular development of young worker bees. Previous studies have confirmed that pollen quality influences ovarian activation [3,4] and the development of hypopharyngeal glands (HGs) [5,6] in honey bee workers.

Pollen diets significantly affect the development of the HGs and ovaries of honey bees [7,8,9]. It is reasonable and reliable to use the degree of HG and ovary development to evaluate pollen quality [10,11]. If workers do not obtain enough nutrition from pollen in early adulthood, hypoplasia of the HG may occur [12]. It has been reported that the ovarian development of worker honey bees can be enhanced by feeding them fresh pollen [11]. Meanwhile, high-protein pollen can promote the development of workers’ ovaries in queenless colonies [13], and low-protein pollen will hinder the development of ovaries [14]. The reward of pollen affects the foraging behavior of honey bee workers.

Flower-naïve honey bees rely heavily on visual cues (color, floral size, patterning, and social cues) and odor cues to discover their first flowers [15,16]. Pollen foraging activity in honey bees is a collective behavior that is regulated by the forager genotype [17], pollen storage level in the hive [18], amount of larvae in the colony [19], and available resources in the environment [20]. The behavior of foragers could be driven by nutritional deficiencies in the colony to maintain the health of colonies [21]. The nutritional composition varies widely in different pollens of different plant species, and not all pollens are equally collected by honey bees [22]. Previous studies have shown that in both natural context and controlled-choice experiments [23], honey bees prefer to collect some pollen types over others [24].

With a large size, rich juice, and sweet, crisp flesh, *Pyrus bretschneideri* Rehd. (Rosales: Rosaceae) is widely cultivated in China [25]. This species experiences a low fruit setting percentage due to self-incompatibility and depends on cross-pollination to set fruit [26]. In the field, pear flowers offer abundant pollen, but *Apis mellifera* workers visit them for a short duration and often switch to other competitively blooming flowers, such as peach, rape, paulownia, and dandelion [27,28]. Compared with pear flowers, apricot (*Armeniaca sibirica*) flowers, which also bloom in early spring, and are more attractive to honey bees.

In this study, to better understand of pollen’s role as a reward and the foraging decision making of workers, we evaluated the preference, consumption, and extraction efficiency of pollen for both pollen diets (pear pollen and apricot pollen) under controlled conditions in the laboratory. We also compared the effect of different pollen diets on the physiological status (the development of HG glands and ovaries) of *A. mellifera* workers.

## 2. Materials and Methods

### 2.1. Bee Rearing and Feeding

This study was conducted during June and August 2020 at the Institute of Apicultural Research, Chinese Academy of Agricultural Sciences (IAR-CAAS). Frames of emerging broods collected from three queenright *A. mellifera* colonies were placed in an incubator at 35 °C and 50% relative humidity. Groups of 80 newly emerged workers were collected and put into wood cages with wire mesh (13 × 14 × 14 cm). The queenless workers were maintained in an incubator at 30 °C and 50% relative humidity. The workers were fed a 50% (*w*/*w*) sucrose solution and supplied with one of two types of pollen pellets, i.e., pear pollen or apricot pollen mixed with sucrose solution. The pollen pellets and sucrose solution were supplied in ELISA plate strips every 24 h. Six cages were set up with 3 cages per treatment. Trapped pollen of both types was collected from colonies to be used for pollen digestibility testing and pollen reference checking. Pear pollen was collected in a large-scale planting orchard in Yuncheng, Shanxi Province. Apricot pollen was purchased from a commercial company. Both types of pollen were collected in the spring of 2020. The purity of pollen was checked under a microscope. Surviving workers were frozen in liquid nitrogen on Day 14, and then stored at −80 °C until dissection.

### 2.2. Pollen Diet Preferences and Consumption

**Figure 1:**
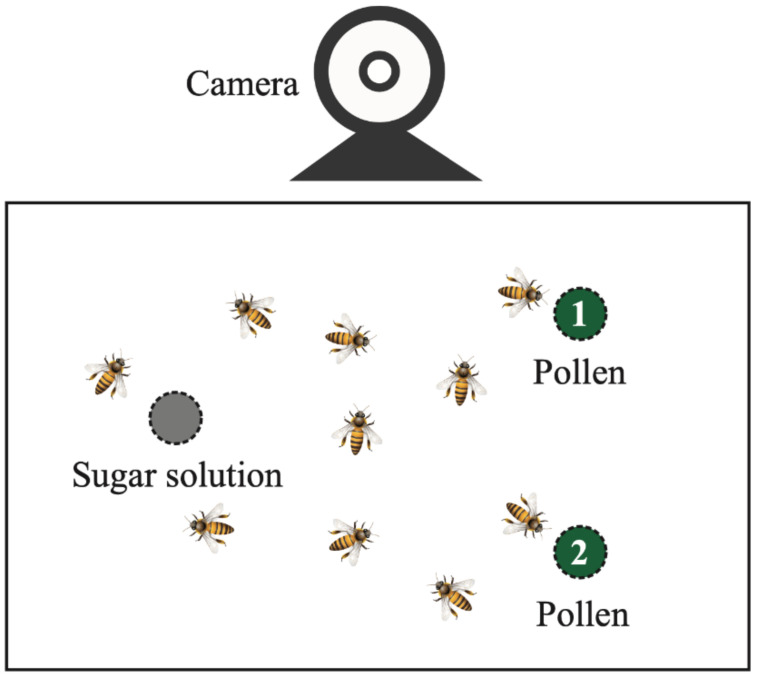
Schematic setup of the dual choice test of pollen diet preference.

A dual choice test was carried out to determine the preference between pear pollen and apricot pollen diets (Figure 1). For each test, a group of 35 workers was collected randomly from one colony and placed into wood cages with wire mesh (13 × 14 × 14 cm). Three cages were prepared and monitored. In each cage, both pear pollen and apricot pollen were supplied at all times at a distance of 5 cm. Sucrose solution (50% *w*/*w*) was supplied at the far end of the cage. Pollen pellets and sucrose solution were replaced every day, and the strips containing the pollen were weighed daily. We measured pollen diet consumption by subtracting the pre-feeding weight from the post-feeding weight [29]. Pollen consumption was recorded daily, and the total amount of pollen pellets that was consumed per day in each cage was compared. The positions of the pollen diets were interchanged to exclude position biases (Figure S1). We used a digital video camera to record the number of workers consuming each pollen diet from 9:00 to 16:00 for 4 continuous days. The number of workers landing on the pollen pellets was counted for 10 min every hour.

### 2.3. Pollen Extraction Efficiency

To measure pollen digestibility, we recorded the extraction efficiency by counting the empty pollen grains in the rectal contents of 20 workers per treatment. The rectal contents of each worker were placed on a slide with a droplet of distilled water, and then stained with cotton lactophenol blue, which stains the cytoplasm dark blue but leaves the cell walls unstained [30]. Then, the mixture was transferred to a hemocytometer, and 100 grains were examined under a phase contrast microscope. For each dissected worker, three counts of 100 grains were recorded. We scored the grains as full, half full, or empty, as defined by Human and Nicolson (Figure S2A) [30]. The stored pear pollen and apricot pollen were used as references to determine the number of initially empty grains for comparison with pollen in the rectum of the honey bees. The following formula was used to calculate the extraction efficiency [31]:

$$\mathrm{Extraction}\;\mathrm{efficiency}\;(\%)\;=\;\frac{No.\;\;of\;empty\;grains-No.\;\;of\;empty\;grains\;in\;fresh\;pollen}{No.\;\;of\;grains}\times 100$$

### 2.4. HG Development

From each cage, thirty workers were randomly selected for HG measurement, with a total of 90 workers representing each pollen diet. The paired HGs removed from the honey bee heads were placed on a glass slide with a droplet of Ringer’s solution and measured under a stereoscopic microscope (Olympus SZX17, Olymplus Corporation, Tokyo, Japan) equipped with a digital camera. The HGs were photographed using DPController (Olympus, Olymplus Corporation, Tokyo, Japan) (Figure S2B,C), and the diameters of acini were measured using ImageJ 1.52a software(NIH Image, Research Services Branch, USA). The development of HGs was determined by measuring the area of acini (mm^2^) with clear borders. For each bee, twenty randomly selected acini per gland were measured. The area of each acinus was calculated according to the following equation [32]:

$$\mathrm{Acini}\;\mathrm{surface}\;\mathrm{area}\;=\;\frac{a\times b}{2}\;\times \;\pi$$

where a is the maximum length, b is the maximum width of the acini, and π = 3.14.

### 2.5. Ovarian Development

Thirty workers from each cage were dissected, yielding a total of 90 bees per treatment. Both ovaries were examined in each bee, and their development was categorized into four stages, according to Hoover et al. (2006) and Tanaka et al. (2019) as follows: Stage I, immature ovary with thread-like or slightly swollen ovaries, egg cells are undistinguished from nutritive cells; Stage II, egg cells are smaller than nutritive cells; Stage III, egg cells are larger than nutritive cells; and Stage IV, well-developed ovaries, with at least one ovariole containing a mature egg cell (Figure S2D–G) [3,33].

## 3. Results

### 3.1. Pollen Diet Preferences and Consumption

**Figure 2:**
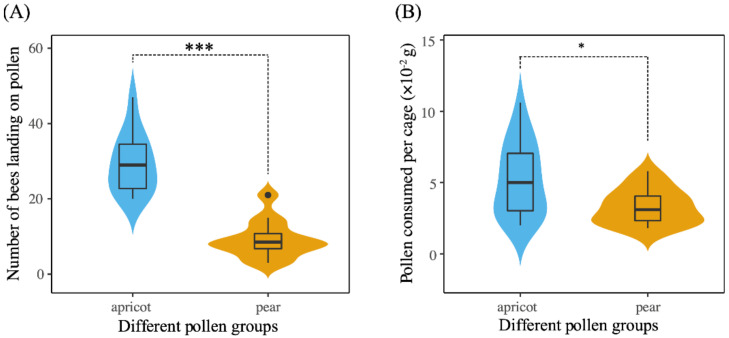
(**A**) Pollen diet preferences and consumption. Number of landings by workers in the dual choice test of apricot pollen and pear pollen diets; (**B**) Consumption of pollen in the dual choice test. Violin plots showing these results; the violin shape shows the distribution of the values, and there is a box plot inside. The black line in the center indicates the median, the top of the black box represents the upper quartile, and the bottom of the black box represents the lower quartile. Asterisks represent the level of significance. * *p* < 0.05 and *** *p* < 0.001.

The number of landings per day made on pear pollen (9.25 ± 5.10) was significantly less than that on apricot pollen (29.5 ± 8.11) (Student’s *t*-test, t = 7.323, df = 22, *p* < 0.001, Figure 2A). The choice tests indicated a significant preference of *A. mellifera* workers for apricot pollen over pear pollen.

In the choice-test cages, workers consumed more apricot pollen (average total amount consumed per day = 0.052 ± 0.026 g per cage) than pear pollen (average total amount consumed per day = 0.033 ± 0.013 mg per cage). Pollen consumption was significantly different between the two pollen diets (Student’s *t*-test, t = 2.404, df = 28, *p* < 0.05, Figure 2B).

### 3.2. Pollen Extraction Efficiency

**Figure 3:**
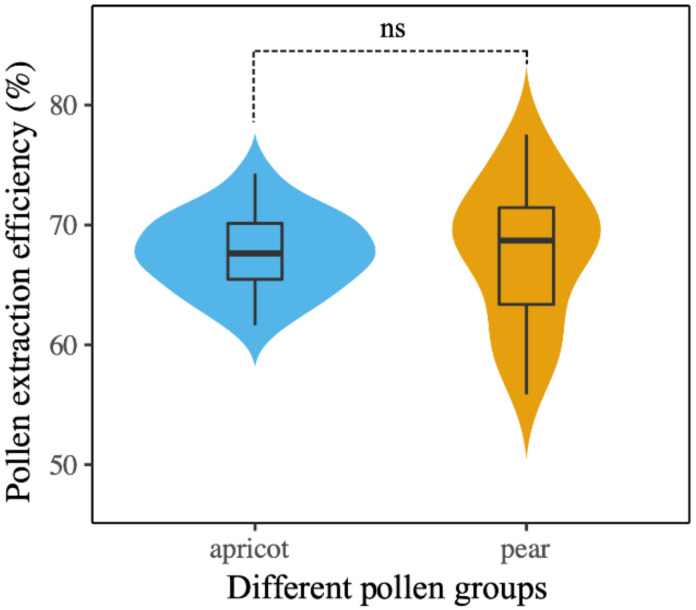
Pollen extraction efficiency for apricot pollen and pear pollen in the rectum of *Apis. mellifera* workers. Violin plots showing these results; the violin shape shows the distribution of the values, and there is a box plot inside. The black line in the center indicates the median, the top of the black box represents the upper quartile, the bottom of the black box represents the lower quartile, and ns means no significance (*p* > 0.05).

The percentages of full, half-full, and empty pollen grains in the reference pollen and rectal contents of workers are shown in Table 1. The percentages of half-full and empty grains were slightly higher in the rectum of workers fed apricot pollen than in those fed pear pollen. The extraction efficiency was 67.63% for pear pollen and 67.73% for apricot pollen. There was no significant difference in the extraction efficiency between these two pollen diets (Student’s *t*-test, t = 0.066, df = 29.545, *p* > 0.05, Figure 3).

### 3.3. HG Development

**Figure 4:**
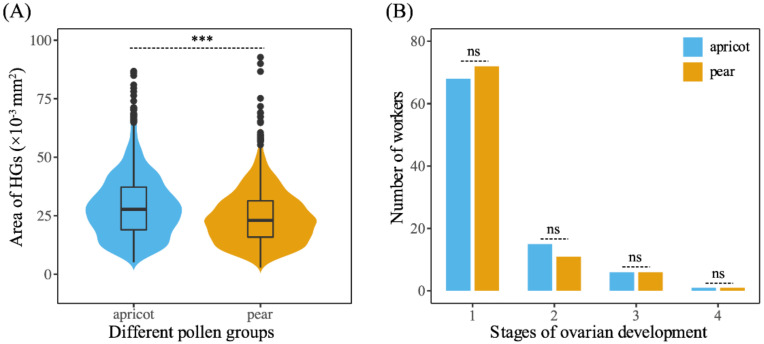
(**A**) Hypopharyngeal gland (HG) development of workers fed apricot pollen and pear pollen diets; (**B**) Ovarian development of workers fed apricot pollen and pear pollen diets. Violin plots showing these results; the violin shape shows the distribution of the values, and there is a box plot inside. The black line in the center indicates the median, the top of the black box represents the upper quartile, the bottom of the black box represents the lower quartile, and the black dots indicate outliers. Asterisks represent the level of significance. *** *p* < 0.001, ns *p* > 0.05.

Workers fed apricot pollen had larger HGs (0.029 ± 0.014 mm^2^) than those fed pear pollen (0.025 ± 0.012 mm^2^). There was a significant difference in HG acinus size between these two pollen diet treatments (Mann–Whitney U test, U = 1299208.5, *p* < 0.001, Figure 4A).

### 3.4. Ovarian Development

The proportion of caged workers showing inactive ovaries was slightly higher for those fed the pear pollen diet than those fed the apricot pollen diet. The number of workers with stage II ovaries was lower in the pear pollen diet treatment group than that in the apricot pollen diet treatment group. Overall, there was no significant difference in the ovarian activation of caged workers aged 14 days supplied with the two different pollen diets (χ^2^ test, χ^2^ = 1.011, df = 3, *p* = 0.849, Figure 4B).

## 4. Discussion

Our experiment demonstrated that workers who had no experience with apricot pollen or pear pollen visited apricot pollen significantly more often than pear pollen in the controlled choice test. A previous study showed that when pollen from different sources was offered simultaneously within the hive, the bees ate more of some types of pollen than others [34]. Our research confirmed that different pollen diets were not equally consumed, and *A. mellifera* workers showed a significantly higher consumption of apricot pollen than pear pollen. This result indicated that bees could discriminate between these two pollen diets and preferred apricot pollen over pear pollen. The bias foraging behavior for apricot pollen is also reflected in the field. However, the substances that elicit pollen-foraging behavior in the field may not act as the same phagostimulant that evokes pollen feeding behavior from caged workers, and this phenomenon needs further investigation.

The protein nutrition of pollen is important for certain aspects of nurse bee physiology, such as the development of the HGs [35] and ovaries [3]. Small HGs could result from a decrease in the availability and protein content of pollen [22,36]. Our study demonstrated that different pollen diets affected the development of HGs differently, with a significantly greater area of acini in caged workers fed apricot pollen diets than in those fed pear pollen diets. This influence may be due to the increased protein intake resulting from the high consumption rate. In addition to protein content, the different effects of pollen diets on HG development could also be explained by other limiting factors, such as amino acids, lipids, and vitamins [37]. The protein content of pollen is a reliable and direct measure of pollen quality [11], and it was suggested as the first criterion for honey bee pollen-foraging preference [38]. However, our primary experiment showed that pear pollen and apricot pollen had comparable protein contents (2699 mg/kg and 2811 mg/kg, Table S1). This result is consistent with the report by Schmidt (1982), who showed that honey bee preference for pollen was not based on its protein content [39]. However, other studies have demonstrated that amino acid composition [40], phenolic contents [41], and protein contents [38] in pollen sources could all affect honey bee foraging preferences. Furthermore, olfactory and chemotactile stimulation also have the potential to influence honey bees’ pollen-foraging behavior [42]. Further studies are needed to compare other qualitative parameter differences to determine what renders pear pollen a nonpreferred diet for honey bees.

Some studies have shown that pear nectar is less abundant and that the sugar concentration in this nectar is low, which makes it less attractive to honey bees and other pollinators [27,43]. During collection, foraging workers normally add regurgitated nectar or honey when packing pollen into their corbicula. This honey may differentially influence the palatability of various pollens to bees [44]. However, this possibility needs to be investigated.

There are still important gaps in our understanding of the ecology and evolution of honey bee and flower interactions. Fundamental questions regarding the adaptive value of floral pollen rewards still remain, and little is known regarding how nutrients in pollen rewards are processed pre- and post-ingestion by honey bees and how reward values turn into foraging decisions [45]. In future studies, to reveal the molecular pathways that are involved, we plan to assess the transcriptional changes in the heads of honey bees fed pear pollen and apricot pollen to compare their encoding differences.

## 5. Conclusions

Pollen is an important source of essential amino acids, minerals, vitamins, and lipids for honey bee larval development. The honey bee makes a decision to collect pollen according to some pollen cues. This means that honey bees tend to collect pollen from some plants over others that bloom at the same time. Clarifying the influence of pollen on the collection behaviors of honey bees will help to improve their pollination service for crops. Our research showed a significant preference of *A. mellifera* workers for apricot pollen diets over pear pollen diets, and apricot pollen promoted the development of workers’ HG significantly more than pear pollen. These results indicated that pear pollen may contain some repellents or exhibit nutritional deficiencies. Therefore, colonies should be supplied with additional pollen when using honey bees for pear tree pollination. However, the repellents and nutritional elements need to be further investigated. Pollens were demonstrated that significantly affected the development of honey bee workers’ HG. These basic research results can be used to evaluate the effects of pollen nutrition on workers.

## Figures and Tables

**Table 1:** Percentages of full, half-full, and empty pollen grains in the reference pollen (before digestion) and the rectal contents of dissected workers (after digestion).

Pollen Type	Full (%)	Half Full (%)	Empty (%)
*Armeniaca. sibirica* pollen/reference	91.70 ± 7.17	5.93 ± 5.19	2.37 ± 2.51
*Armeniaca. sibirica* pollen/rectum	5.53 ± 3.11	24.37 ± 5.04	70.00 ± 5.70
*Pyrus. bretschneideri* pollen/reference	93.30 ± 3.01	5.23 ± 2.81	1.47 ± 1.28
*Pyrus. bretschneideri* pollen/rectum	9.20 ± 5.33	21.70 ± 6.96	69.10 ± 7.79

## Data Availability

Data are contained within the article or supplementary material.

## Supplementary Materials

The following are available online at https://www.mdpi.com/2075-4450/12/2/130/s1, Figure S1: Number of landings by workers in the dual choice test of apricot pollen (left column group) and pear pollen (right column group) diets. ns represents the level of significance, *p* > 0.05. Figure S2: Photos of pollen extraction efficiency, HG and ovary development. (A) Pollen of honey bee’s hindgut that fed with apricot pollen. Grains were stained with cotton lactophenol blue. (a) Full pollen grain, (b) Half-full pollen grain, (c) Empty pollen grain. (B) HG of honey bee fed with pear pollen. (C) HG of honey bee fed with apricot pollen. (D–G) Four development stages of ovary (I–IV). Table S1: List of main compounds of apricot and pear pollen.

## References

[1] Dotterl S.D., Vereecken N.J.V.J. (2010). The chemical ecology and evolution of bee–flower interactions: A review and perspectivesThe present review is one in the special series of reviews on animal–plant interactions. Can. J. Zool..

[2] Brodschneider R., Crailsheim K. (2010). Nutrition and health in honey bees. Apidologie.

[3] Hoover S.E., Higo H.A., Winston M.L. (2005). Worker honey bee ovary development: Seasonal variation and the influence of larval and adult nutrition. J. Comp. Physiol. B.

[4] Nicolson S.W., Neves S.D.S.D., Human H., Pirk C.W.W. (2018). Digestibility and nutritional value of fresh and stored pollen for honey bees (*Apis mellifera* scutellata). J. Insect Physiol..

[5] Standifer L.N. (1967). A comparison of the protein quality of pollens for growth-stimulation of the hypopharyngeal glands and longevity of honey bees, *Apis mellifera* L. (Hymenoptera: Apidae). Insectes Sociaux.

[6] Di Pasquale G., Alaux C., Le Conte Y., Odoux J.-F., Pioz M., Vaissière B.E., Belzunces L.P., Decourtye A. (2016). Variations in the Availability of Pollen Resources Affect Honey Bee Health. PLoS ONE.

[7] Corby-Harris V., Snyder L., Meador C. (2019). Fat body lipolysis connects poor nutrition to hypopharyngeal gland degradation in *Apis mellifera*. J. Insect Physiol..

[8] Al-Ghamdi A.A., Al-Khaibari A.M., Omar M.O. (2011). Consumption rate of some proteinic diets affecting hypopharyngeal glands development in honeybee workers. Saudi J. Biol. Sci..

[9] Human H., Nicolson S.W., Strauss K., Pirk C.W.W., Dietemann V. (2007). Influence of pollen quality on ovarian development in honeybee workers (*Apis mellifera* scutellata). J. Insect Physiol..

[10] Hrassnigg N., Crailsheim K. (1998). Adaptation of hypopharyngeal gland development to the brood status of honeybee (*Apis mellifera* L.) colonies. J. Insect Physiol..

[11] Pernal S.F., Currie R.W. (2000). Pollen quality of fresh and 1-year-old single pollen diets for worker honey bees (*Apis mellifera* L.). Apidologie.

[12] Corby-Harris V., Meador C.A., Snyder L.A., Schwan M.R., Maes P., Jones B.M., Walton A., Anderson K.E. (2016). Transcriptional, translational, and physiological signatures of undernourished honey bees (*Apis mellifera*) suggest a role for hormonal factors in hypopharyngeal gland degradation. J. Insect Physiol..

[13] Jay S. (1993). The effect of kiwifruit (Actinidia deliciosa A Chev) and yellow flowered broom (*Cytisus scoparius* Link) pollen on the ovary development of worker honey bees (*Apis mellifera* L). Apidologie.

[14] Harris J.W., Harbo J.R. (1990). Suppression of Ovary Development of Worker Honeybees by Association with Workers Treated with Carbon Dioxide. J. Apic. Res..

[15] Riffell J.A. (2011). The Neuroecology of a Pollinator’s Buffet: Olfactory Preferences and Learning in Insect Pollinators. Integr. Comp. Biol..

[16] Orbán L.L., Plowright C.M.S. (2014). Getting to the start line: How bumblebees and honeybees are visually guided towards their first floral contact. Insectes Sociaux.

[17] Waddington K.D., Nelson C., Page R.E. (1998). Effects of pollen quality and genotype on the dance of foraging honey bees. Anim. Behav..

[18] Weidenmüller A., Tautz J. (2002). In-Hive Behavior of Pollen Foragers (*Apis mellifera*) in Honey Bee Colonies under Conditions of High and Low Pollen Need. Ethology.

[19] Fewell J.H., Winston M.L. (1992). Colony state and regulation of pollen foraging in the honey bee, *Apis mellifera* L.. Behav. Ecol. Sociobiol..

[20] Beekman M., Gilchrist A.L., Duncan M., Sumpter D.J.T. (2007). What makes a honeybee scout?. Behav. Ecol. Sociobiol..

[21] Hendriksma H., Shafir S. (2016). Honey bee foragers balance colony nutritional deficiencies. Behav. Ecol. Sociobiol..

[22] Di Pasquale G., Salignon M., Le Conte Y., Belzunces L.P., Decourtye A., Kretzschmar A., Suchail S., Brunet J.-L., Alaux C. (2013). Influence of Pollen Nutrition on Honey Bee Health: Do Pollen Quality and Diversity Matter?. PLoS ONE.

[23] Boelter A., Wilson W. (1984). Attempts to condition the pollen preference of honey bees. Am. Bee J..

[24] Yang H., Sun J., Tang P., Ma C., Luo S., Wu J. (2020). The Ratio of Sunflower Pollens Foraged by *Apis mellifera* Is More Than That of Apis cerana does during Sunflower Blooming. Sociobiology.

[25] Qin G., Tao S., Zhang H., Huang W., Wu J., Xu Y., Zhang S. (2014). Evolution of the Aroma Volatiles of Pear Fruits Supplemented with Fatty Acid Metabolic Precursors. Molecules.

[26] Hiratsuka S., Zhang S.-L. (2002). Relationships between fruit set, pollen-tube growth, and S-RNase concentration in the self-incompatible Japanese pear. Sci. Hortic..

[27] Gemeda T.K., Shao Y., Wu W., Yang H., Huang J., Wu J. (2017). Native Honey Bees Outperform Adventive Honey Bees in Increasing *Pyrus bretschneideri* (Rosales: Rosaceae) Pollination. J. Econ. Entomol..

[28] Gemeda T.K., Li J., Luo S., Yang H., Jin T., Huang J., Wu J. (2018). Pollen trapping and sugar syrup feeding of honey bee (Hymenoptera: Apidae) enhance pollen collection of less preferred flowers. PLoS ONE.

[29] Peters L., Zhu-Salzman K., Pankiw T. (2010). Effect of primer pheromones and pollen diet on the food producing glands of worker honey bees (*Apis mellifera* L.). J. Insect Physiol..

[30] Human H., Nicolson S.W. (2003). Digestion of maize and sunflower pollen by the spotted maize beetle *Astylus atromaculatus* (Melyridae): Is there a role for osmotic shock?. J. Insect Physiol..

[31] Brice A.T., Dahl K.H., Grau C.R. (1989). Pollen Digestibility by Hummingbirds and Psittacines. Condor.

[32] Lee M.R., Choi Y.S., Kim D.W., Lee M.Y. (2019). Age-dependent hypopharyngeal gland development and morphometric characteristics in the cross-bred lineage of honeybees reared for high royal jelly production. J. Asia Pac. Entomol..

[33] Tanaka C.S., Ikemoto M., Nikkeshi A., Kanbe Y., Mitsuhata M., Yokoi T. (2019). Ovarian development related to pollen feeding in workers of the bumblebee *Bombus ignitus* (Hymenoptera: Apidae). Appl. Entomol. Zooɭ..

[34] Campana B.J., Moeller F.E. (1977). Honey Bees: Preference for and Nutritive Value of Pollen from Five Plant Sources123. J. Econ. Entomol..

[35] Crailsheim K., Stolberg E. (1989). Influence of diet, age and colony condition upon intestinal proteolytic activity and size of the hypopharyngeal glands in the honeybee (*Apis mellifera* L.). J. Insect Physiol..

[36] DeGrandi-Hoffman G., Chen Y., Huang E., Huang M.H. (2010). The effect of diet on protein concentration, hypopharyngeal gland development and virus load in worker honey bees (*Apis mellifera* L.). J. Insect Physiol..

[37] Omar E., Abd-Ella A., Khodairy M.M., Moosbeckhofer R., Crailsheim K., Brodschneider R. (2016). Influence of different pollen diets on the development of hypopharyngeal glands and size of acid gland sacs in caged honey bees (*Apis mellifera*). Apidologie.

[38] Ghosh S., Jeon H., Jung C. (2020). Foraging behaviour and preference of pollen sources by honey bee (*Apis mellifera*) relative to protein contents. J. Ecol. Environ..

[39] Schmidt J.O. (1982). Pollen foraging preferences of honey bees. Southwest. Entomol..

[40] Cook S.M., Awmack C.S., Murray D.A., Williams I. (2003). Are honey bees’ foraging preferences affected by pollen amino acid composition?. Ecol. Entomol..

[41] Liu F., Zhang X.-W., Chai J.-P., Yang D.-R. (2005). Pollen phenolics and regulation of pollen foraging in honeybee colony. Behav. Ecol. Sociobiol..

[42] Ruedenauer F.A., Wöhrle C., Spaethe J., Leonhardt S.D. (2018). Do honeybees (*Apis mellifera*) differentiate between different pollen types?. PLoS ONE.

[43] Smessaert J., Honnay O., Keulemans W. (2019). Monitoring pollinator activity in an apple and pear orchard, linked with the analysis of the nectar composition. Acta Hortic..

[44] Boch R. (1982). Relative Attractiveness of Different Pollens to Honeybees when Foraging in a Flight Room and when Fed in the Hive. J. Apic. Res..

[45] Nicholls E., De Ibarra N.H. (2016). Assessment of pollen rewards by foraging bees. Funct. Ecol..

