# Supplementary material for: Pollen Source Affects Development and Behavioral Preferences in Honey Bees
Source: Insects. 2021 Feb 2;12(2):130. doi: 10.3390/insects12020130 (PMC7913020; doi:10.3390/insects12020130)
Supplement: Supplementary file 1 [file insects-12-00130-s001.pdf]

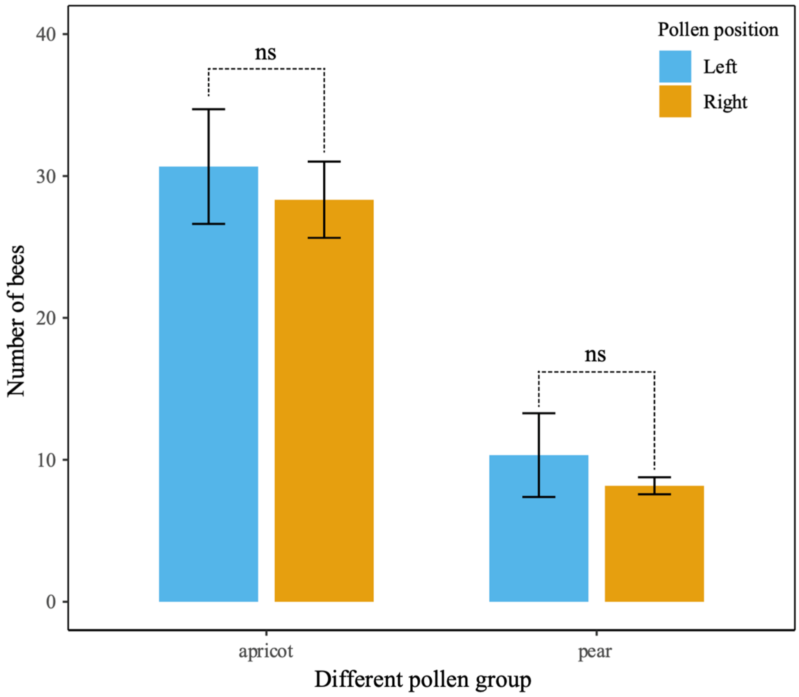

Figure S1. Number of landings by workers in the dual choice test of apricot pollen (left column group) and pear pollen (right column group) diets. ns represents the level of significance, $p > 0.05$.

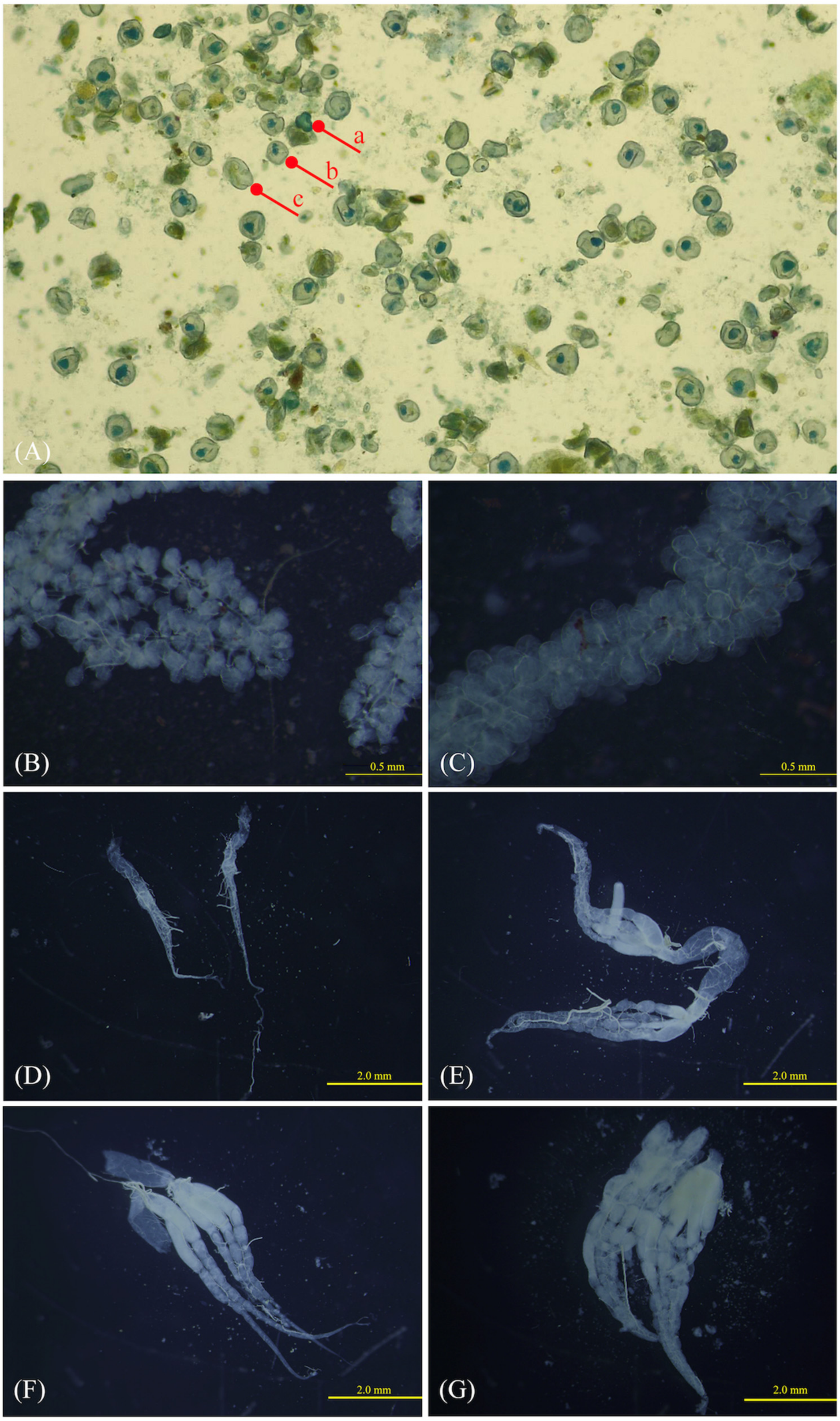

Figure S2. Photos of pollen extraction efficiency, HG and ovary development. (**A**) Pollen of honey bee's hindgut that fed with apricot pollen. Grains were stained with cotton lactophenol blue. (a) Full pollen grain, (b) half-full pollen grain, (c) Empty pollen grain. (**B**) HG of honey bee fed with pear pollen. (**C**) HG of honey bee fed with apricot pollen. (**D**–**G**) Four development stages of ovary (**I**–**IV**).

Table S1. List of main compounds of apricot and pear pollen.

ID	Compound	Apricot	Pear
1	Protein	2811	2699
2	Fat	250	90
3	Threonine	35.8	109.7
4	Valine	53.9	52.7
5	Methionine	1.2	7.4
6	Isoleucine	11.7	27.6
7	Leucine	11.1	18.6
8	Phenylalanine	18.4	99.6
9	Histidine	160.8	1041.8
10	Lysine	20.7	91.5
11	Arginine	3254	5492
12	Tryptophan	60.8	24.2

*The unit is mg/kg.
